# Perception of feeling safe perioperatively: a concept analysis

**DOI:** 10.1080/17482631.2023.2216018

**Published:** 2023-05-21

**Authors:** Fanny Larsson, Ulrica Strömbäck, Silje Rysst Gustafsson, Åsa Engström

**Affiliations:** Division of Nursing and Medical Technology, Department of Health, Education and Technology, Luleå University of Technology, Luleå, Sweden

**Keywords:** Feeling safe, perioperative, concept analysis, nursing, literature review

## Abstract

**Purpose:**

The purpose of this study was to explore the concept of feeling safe, from the patient perspective, in a perioperative context.

**Method:**

The eight-step concept analysis approach proposed by Walker and Avant was utilized to examine the attributes of feeling safe. Uses of the concept, defining attributes as well as antecedents, consequences and empirical referents are presented to describe the concept. Case examples are provided in order to assist the understanding of the defining attributes.

**Results:**

Feeling safe is defined as: a person that does not feel worried or threatened. Three attributes were identified: Participation, Control and Presence. Knowledge and Relationship are the antecedents of feeling safe, while Feeling Acknowledged and Trust are the consequences. Empirical referents are explored in order to find a way to measuring the perceived feeling of safety.

**Conclusion:**

This concept analysis underscores the importance of including patients’ perceptions in traditional patient safety work. Patients who feel safe perceive that they participate in their care, that they are in control, and that they feel the presence of both healthcare staff and relatives. The perceived feeling of security could, by extension, promote the postoperative recovery of patients undergoing surgery by positively affect the process of recovery.

## Introduction

For patients, the perceived feeling of safety may be just as important as the fact of actually being safe throughout a procedure (Lasiter, 2011; Mollon, 2014; Péculo-Carrasco et al., 2020). A perceived feeling of safety is influenced by a number of things, including person-centred care and information (Péculo-Carrasco et al., 2020). Vulnerability, frustration, and anxiety are some feelings that can arise when a person feels unsafe; consequently, due to difficulty in relaxing, the healing process can be more arduous (Mollon, 2014; Wassenaar et al., 2014). In the postoperative period, patients’ perceptions of feeling safe can have an impact on the postoperative recovery. Feeling safe is achieved when one is treated as an individual and feels reassured during recovery (Dahlberg et al., 2018). However, much of today’s safety-related research focuses on the delivery of safe care, and little research is done on the concept of feeling safe and what it entails from the patient’s perspective (Mollon, 2014).

The concept of feeling safe in nursing has been described by Segesten (1984), who found that feeling safe is consistent with feeling at peace and feeling out of danger. Every person has a desire to feel safe, but the feeling of security varies from person to person. Segesten’s (1984) definition of the concept consists of two dimensions: internal safety and external safety. The internal dimension is linked to early childhood experiences and continues to grow throughout one’s life; it is described as a feeling of warmth, harmony, calm, and trust, and it includes the ability to believe in oneself and confess one’s flaws. The external dimension of feeling safe is comprised of knowledge, control, and trustworthy individuals, and it is influenced by the person’s surroundings (Segesten, 1984). Mollon (2014) has studied the concept of feeling safe for hospitalized patients, and she concludes that the concept in that specific context consists of four attributes: (1) *feeling trust*, (2) *feeling cared for*, (3) *presence of another human being*, and (4) *knowledge*.

Patient safety can be defined as the absence of unintentional injury (Donaldson et al., 2000). The World Health Organization (WHO) has recognized patient safety as a priority in health care through their acceptance of the resolution “Global action on patient safety” (World Health Organization, n.d.). The care that is given should meet the patient’s needs for security, continuity, and desire to feel safe (The National Board of Health and Welfare, 2022). Nurses play a crucial part in patients’ feelings of safety (Lovink et al., 2015). According to the Nursing Code of Ethics by American Nurses Association (ANA) the promotion, advocation and protection of patients’ rights, health and safety is a part of a nurse’s ethical duty (American Nurses Association, 2015). Two of the core competencies for Swedish registered nurse anesthetists, are person-centred care and safe care; these include (among other things) the requirement to offer safe care that supports patients’ sense of security and trust (Riksföreningen för anestesi och intensivvård & Svensk sjuksköterskeförening, 2020). With more complex health care, the risk of an adverse incident increases (Oregas et al., 2008). The perioperative context is complex and highly technical, and the environment differs from other health care settings; nevertheless, nurses working in a perioperative setting need to provide care that supports patients’ individual needs, even if the environment is fast-paced and filled with challenging situations (Spruce, 2013). Moreover, nurses working in a perioperative setting need to establish a relationship with their patients based on a meeting in a brief period of time (Lekens et al., 2023). Given the complexity that is associated with a perioperative context, a focus on patient safety and patient perception of feeling safe is crucial.

In summary, the practice of providing a patient with safe care that meets their need to feel safe—in addition to the parts that more directly focus on delivering care that prevents and minimizes risk, mistakes, and injury to patients—is an important part of perioperative nursing. Even so, there is a lack of clarity as to what the concept of “feeling safe” means in a perioperative setting. Therefore, the purpose of this study was to explore the concept of feeling safe, from the patient perspective, in a perioperative context.

## Material and methods

The concept analysis approach proposed by Walker and Avant (2011) was utilized to examine the attributes of feeling safe in a perioperative context. Walker and Avant (2011) shortened Wilson’s (1963) classic eleven-step concept analysis approach to eight steps; the latter is summarized in Table 1.

**Table 1.** Walker and Avant (2011) eight-step procedure.

1	Selecting a concept
2	Determining the aim of the analysis
3	Identifying all uses of the concept
4	Determining the defining attributes
5	Identifying a model case of the concept
6	Identifying additional cases
7	Identifying antecedents and consequences
8	Defining empirical referents

A concept analysis looks at the structure and function of a concept, which must be investigated in order to have a better understanding of the phenomena being examined (Walker & Avant, 2011).

## Data sources

An initial scoping search was conducted in 2022, using the keyword “feeling safe” to scan relevant articles for surrogate terms relevant to the concept. Through this process, the following additional keywords were identified: feel safe, feeling of safety, feeling secure, feeling of security, sense of safety, and sense of security. The scoping search was followed by a systematic literature search of the PsycInfo, CINAHL, and Medline databases. The purpose of the literature search was to identify as many available peer-reviewed scholarly papers as possible that use the concept of feeling safe in a perioperative setting. Articles were not excluded by a time limit, because one of the aims of this analysis was to identify all uses of the concept. The search comprised the different terms for feeling safe that were mentioned above, in combination with various terms for a perioperative context, such as perioperative, surgery, operation, etc. The Boolean operators AND and OR were used to combine keywords. The search strategy is displayed in Table 2.

**Table 2.** Search strategy.

Query	Limiters/expanders			Results
**Cinahl 080322 Limitations: Peer Review, English language**				
S1	“feeling safe”			244
S2	“feeling secure”			62
S3	“feeling of safety”			72
S4	“feeling of security”			52
S5	“feel* safe*”			622
S6	“feel* secur*”			139
S7	“sense* of safe*”			263
S8	“sense* of secur*”			571
S9	S1 OR S2 OR S3 OR S4 OR S5 OR S6 OR S7 OR S8			1,664
S10	Preoperative*			80,350
S11	Perioperative*			49,192
S12	Peroperative*			283
S13	Intraoperative*			45,721
S14	Postoperative*			196,680
S15	Anesthesia			68,874
S16	Anaesthesia			71,666
S17	Anaesthes*			189,56
S18	Anesthes*			72,533
S19	Surgery			543,644
S20	(MH “Surgery, Operative+”)			620,889
S21	(MH “Anesthesia”			10,891
S22	S10 OR S11 OR S12 OR S13 OR S14 OR S15 OR S16 OR S17 OR S18 OR S19 OR S20 OR S21			898,982
S23	S9 AND S22			95
**PsycInfo 080322, Limitations: Peer reviewed, English**				
Query	Limiters/expanders			Results
S1	“feeling safe”			347
S2	“feeling secure”			64
S3	“feeling of safety”			119
S4	“feeling of security”			182
S5	“feel* safe*”			1,153
S6	“feel* secur*”			271
S7	“sense* of safe*”			593
S8	“sense* of secur*”			828
S9	S1 OR S2 OR S3 OR S4 OR S5 OR S6 OR S7 OR S8			3,047
S10	Preoperative*			5,539
S11	Perioperative*			2,565
S12	Peroperative*			10
S13	Intraoperative*			1,463
S14	Postoperative*			12,064
S15	Anesthesia			11,374
S16	Anaesthesia			11,464
S17	Anaesthes*			3,751
S18	Anesthes*			19,889
S19	Surgery			56,018
S20	S10 OR S11 OR S12 OR S13 OR S14 OR S15 OR S16 OR S17 OR S18 OR S19			80,570
S21	S9 AND S20			40
**Medline 080322, Limitations: Scholarly journals, English language**				
Query	Limiters/expanders			Results
S1	“feeling safe”			326
S2	“feeling secure”			77
S3	“feeling of safety”			154
S4	“feeling of security”			139
S5	“feel* safe*”			929
S6	“feel* secur*”			223
S7	“sense* of safe*”			350
S8	“sense* of secur*”			921
S9	S1 OR S2 OR S3 OR S4 OR S5 OR S6 OR S7 OR S8			2,644
S10	Preoperative*			361,871
S11	Perioperative*			144,450
S12	Peroperative*			4,523
S13	Intraoperative*			201,714
S14	Postoperative*			924,763
S15	Anesthesia			345,160
S16	Anaesthesia			190,760
S17	Anaesthes*			194,481
S18	Anesthes*			517,462
S19	(MH “Anesthesia+”)			200,057
S20	Surgery			399,6171
S21	S10 OR S11 OR S12 OR S13 OR S14 OR S15 OR S16 OR S17 OR S18 OR S19 OR S21			4,576,311
S22	S10 AND S21			255

**Figure 1.**
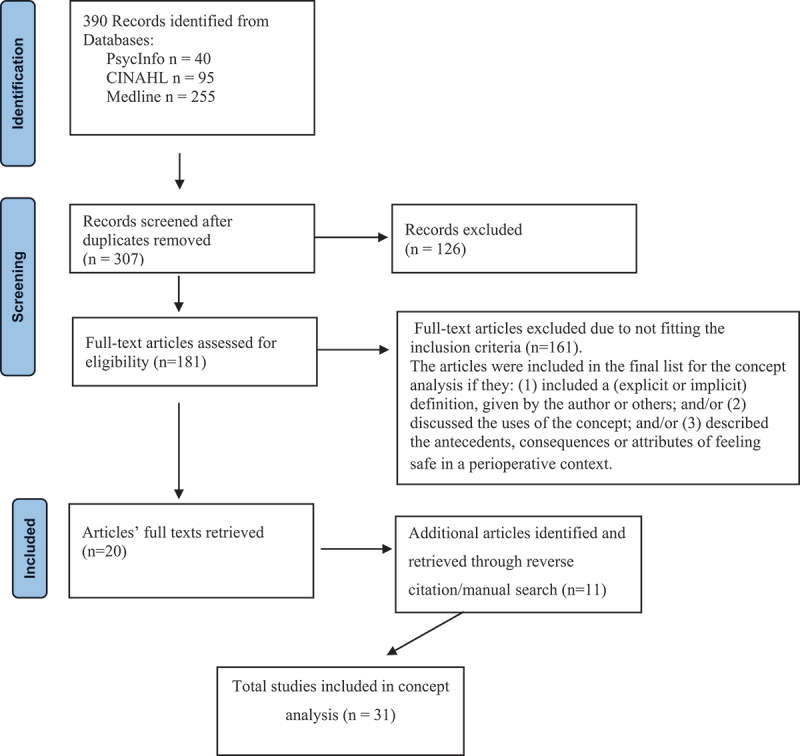
Flowchart of systematic literature search.

The retrieved articles (*n* = 390) were reviewed in terms of their use of the concept of feeling safe in a perioperative setting. After duplicates were removed, a total of 307 articles were screened, and articles that clearly did not fit the inclusion criteria were removed, leaving 181 articles. The full texts of these articles were retrieved and read to determine whether they met the inclusion criteria. The quality of the papers was not assessed using a formal approach, but each of the articles was critically examined in accordance with the inclusion criteria. The articles were included in the final list for the concept analysis if they: (1) included a (explicit or implicit) definition, given by the author or others; and/or (2) discussed the uses of the concept; and/or (3) described the antecedents, consequences, or attributes of feeling safe in a perioperative context. This process led to the exclusion of 161 articles. The reference lists of the retrieved articles were reviewed to find more literature, leading to the inclusion of an additional 11 articles. In total, the analysis comprised 31 articles that met the inclusion criteria. A flow chart in accordance with PRISMA (Page et al., 2021) that illustrates the systematic literature search can be seen in Figure 1. Dictionaries, thesauruses, and literature were searched in order to identify uses of the concept.

From the articles, included characteristics that were used in relation to the concept of feeling safe were extracted; these include such characteristics as participation, autonomy, comprehensive information, and control. In subsequent steps, these characteristics were organized and grouped into categories, which shared similar characteristics. Three categories were chosen as essential attributes of feeling safe.

## Results

### Uses of the concept

The goal of defining the uses of a concept is to find as many uses of the concept as possible, both ordinary and scientific (Walker & Avant, 2011). Dictionaries (Nationalencyklopedin, n.d.; Oxford English Dictionary, 2021a; Oxford English Dictionary, 2021b; Svenska akademins ordböcker, 2021; Merriam-Webster, n.d.a.; Merriam-Webster, n.d.b.) were used to create a foundation by defining the words “feeling” and “safe”, first independently and then as a phrase together. Additional healthcare literature was scanned to find definitions of the phrase feeling safe.

### Feeling

The word “feel” originates from the Germanic (Oxford English Dictionary, 2021a) and is defined as:

1. “To have a sensation, impression, perception, or emotion” (Oxford English Dictionary, 2021a).
2. “To believe, judge, have an opinion” (Oxford English Dictionary, 2021a).
3. “To examine or explore by touch” (Oxford English Dictionary, 2021a).

The word “feeling” was first used in the 12^th^ century with the defined meaning of: “one of the basic physical senses of which the skin contains the chief end organs and of which the sensations of touch and temperature are characteristic” (Webster, n.d.a). The definition of the word “feeling” includes:

1. “An emotional state or reaction” (Webster, n.d.a).
2. “Generalized bodily consciousness or sensation” (Webster, n.d.a).
3. “The undifferentiated background of one’s awareness considered apart from any identifiable sensation, perception, or thought” (Webster, n.d.a).

### Safe

The word “safe” originates from the French word *salf* or *salve* (Oxford English Dictionary, 2021b) and the Latin word *salvus*; it was first used in the 14^th^ century (Webster, n.d.b). It is defined as:

1. “Free from hurt or damage; unharmed” (Oxford English Dictionary, 2021b).
2. “Free from danger; secure” (Oxford English Dictionary, 2021b).
3. “Free from harm or risk: unhurt” (Webster, n.d.b).

In Swedish there is a distinction between the word *säker*, which is defined as being out of danger (Nationalencyklopedin, n.d.), and the word *trygg*, which may be defined in the same way as *säker* but also as a person that does not feel worried or threatened (Svenska akademins ordböcker, 2021).

### Feeling safe

The phrase “feeling safe” has not been found in any dictionaries. The phrase “feeling safe” in the context of care has been defined in various ways, including: (1) a feeling of peace and out of danger (Segesten, 1984); (2) “an emotional state where perceptions of care contribute to a sense of security and freedom from harm” (Mollon, 2014, 1729); and (3) freedom from both physical and emotional threats (Lovink et al., 2015). Psychological safety is a related concept that has been described in different areas. In management, psychological safety has been defined as “feeling able to show and employ one’s self without fear of negative consequences to self-image, status, or career” (Kahn, 1990, p. 708). In a healthcare setting, psychological safety has been defined as “a multilevel phenomenon related to a unit culture that facilitates interpersonal risk behaviour” (Ito et al., 2021, p. 471). Another related concept is that of confidence; Haavardsholmen and Nåden (2009) relate the concept of confidence to Segesten’s work mentioned above and conclude that the concept of confidence is linked to feelings of trust, assurance, and self-reliance.

For the purpose of this paper, “feeling safe” is defined in accordance with the definition of the Swedish word *trygghet*: a person that does not feel worried or threatened (Svenska akademins ordböcker, 2021). This is in line with the definitions of feeling safe provided by Lovink et al. (2015), Mollon (2014), and Segesten (1984).

### Defining attributes

In defining attributes of a certain concept, the goal is to define the characteristics associated with that concept as well as those that distinguish the specific concept from other, related concepts (Walker & Avant, 2011). In this analysis, the final attributes that define the concept of feeling safe from the patients perspective are Participation, Control, and Presence (Table 3).

**Table 3.** Attributes of feeling safe in perioperative setting.

Source	Number of attributes	Participation	Control	Presence
1. Aasa et al. (2013)	3 of 3	+	+	+
2. Adelani and Barrack (2019)	1 of 3	-	+	-
21 Barthelsson et al. (2003)	1 of 3	-	+	-
3. Bergman et al. (2012)	3 of 3	+	+	+
22 Causey-Upton and Howell (2017)	2 of 3	-	+	+
23 Costa (2001)	2 of 3	+	-	+
4 Cudré et al. (2015)	2 of 3	+	+	-
5 Davis et al. (2013)	1 of 3	-	+	-
6 Dixon et al. (2015)	2 of 3	+	+	-
7 Engström et al. (2017)	3 of 3	+	+	+
8 Forsberg et al. (2014)	2 of 3	-	+	+
24 Forsberg et al. (2015)	1 of 3	-	+	-
9 Gejervall et al. (2007)	1 of 3	-	+	-
25 Gustafsson et al. (2010)	2 of 3	+	+	-
10 Haapala et al. (2013)	2 of 3	+	+	-
11 Heine et al. (2004)	2 of 3	-	+	+
12 Hestdal and Skorpen (2020)	3 of 3	+	+	+
13 Hommel et al. (2012)	2 of 3	-	+	+
14 Høvik et al. (2018)	1 of 3	-	+	-
15 Kaptain et al. (2019)	2 of 3	+	-	+
26 Karlsson et al. (2012)	3 of 3	+	+	+
27 Larsson et al. (2022)	2 of 3	+	+	-
28 Lindwall et al. (2003)	3 of 3	+	+	+
16 Lingehall et al. (2015)	3 of 3	+	+	+
17 Lupieri et al. (2016)	1 of 3	-	+	-
29 Mako et al. (2016)	3 of 3	+	+	+
30 Mauleon et al. (2007)	1 of 3	-	+	-
31 Renholm et al. (2014)	2 of 3	-	+	+
18 Rosén et al. (2008)	1 of 3	-	+	-
19 Tosuner Akpinar et al. (2019)	1 of 3	-	+	-
20 Turesson et al. (2019)	1 of 3	-	+	-

### Participation

Participation and inclusion in care and decision-making (Aasa et al., 2013; Cudré et al., 2015; Gustafsson et al., 2010; Haapala et al., 2013) was the most frequently used characteristic when describing feeling safe in a perioperative context. It is important for patients to be able to affect their own care, and staff should take patients’ needs and considerations into account and invite them to engage in a dialogue (Cudré et al., 2015; Gustafsson et al., 2010; Haapala et al., 2013; Heine et al., 2004; Karlsson et al., 2012; Lindwall et al., 2003; Mako et al., 2016). Furthermore, it is important to be spoken to directly (Engström et al., 2017) and to be seen as a person who is capable and equal, with individual needs, and not just as a patient (Gustafsson et al., 2010; Hestdal & Skorpen, 2020; Kaptain et al., 2019; Karlsson et al., 2012; Lingehall et al., 2015; Renholm et al., 2014).

### Control

The second attribute of feeling safe is Control, which includes ability to trust the staff, their abilities, and competence, as well as to feel like that the staff are in control and supportive and that they are looking after the patient (Aasa et al., 2013; Bergman et al., 2012; Cudré et al., 2015; Dixon et al., 2015; Engström et al., 2017; Forsberg et al., 2014; Gejervall et al., 2007; Gustafsson et al., 2010; Hommel et al., 2012; Kaptain et al., 2019; Karlsson et al., 2012; Lindwall et al., 2003; Lingehall et al., 2015; Mako et al., 2016; Mauleon et al., 2007; Turesson et al., 2019). During surgery, when patients cede control of their body to the staff, there is a risk that the patient feels insecure and has a fear of being abandoned (Dixon et al., 2015; Karlsson et al., 2012). On the personal level, patients have described that it is important to be in control (Forsberg et al., 2014), One way of enabling patients to stay in control may be to have regional anaesthesia, in order for patients to maintain awareness during the procedure (Forsberg et al., 2014); some patients have also described a wish to stay in control by following the procedure from the monitor (Haapala et al., 2020). When patients feel worried and thus less in control, feelings of insecurity arise (Haapala et al., 2013).

### Presence

The last attribute of feeling safe in a perioperative setting is Presence, and it can mean both presence of family and presence of staff. Presence of family members can help patients discuss questions that might arise (Aasa et al., 2013) as well as help patients take part in the information provided (Mako et al., 2016); it could also make patients feel less lonely (Hestdal & Skorpen, 2020). Presence of staff can help patients feel that they are not left alone (Causey-Upton & Howell, 2017; Hestdal & Skorpen, 2020; Lindwall et al., 2003). If patients have eye contact with or physical closeness to the staff, it helps them understand that they are able to make contact when needed, it provides an opportunity to be confirmed, and it shows the staff are a resource for the patient Bergman et al. (2012); (Forsberg et al., 2014; Heine et al., 2004; Hestdal & Skorpen, 2020; Karlsson et al., 2012; Mako et al., 2016). If staff are perceived as stressed or having a negative attitude, or if the staff are not physically close to the patient, the patient might feel insecure about who will respond if they call for attention, and their sense of safety might decrease (Engström et al., 2017; Karlsson et al., 2012).

### Constructed cases

Constructed cases are illustrations of the concept and its defining attributes. Multiple types of constructed cases are used to fully depict the concept. First, a model case that describes all defining attributes is constructed. Then, additional cases—including a borderline case, which describes a case with most (but not all) of the attributes, a related case are related to the concept but do not contain all of the defining attributes, and a contrary case, which describes a case where the attributes are absent—are constructed (Walker & Avant, 2011). These constructed cases may be helpful in attempts to understand what “feeling safe” does and does not entail. To illustrate the concept’s attributes in real-life situations, the following sections discuss a model case, a borderline case, a related case, and a contrary case, which are based on the authors’ previous clinical experiences and examples available in the literature.

### Model case

Daniel is having knee replacement surgery. Unlike the routine procedure, Daniel’s surgery is scheduled to be performed as a day surgery. Together with his husband, Daniel attended a preoperative appointment where he met the surgeon, a nurse, and a physiotherapist. At the appointment, the couple were provided with information, both orally and written, about the procedure, the postoperative period, and what Daniel might expect in terms of pain and other complications that might occur during the recovery process. He also received information about what it means to undergo this procedure as a day surgery and had the opportunity to ask questions. On the day of the surgery, Daniel meets the surgeon and has the opportunity to ask question that have arisen since the appointment. The staff at the unit speaks directly to him and provides him with information on what is about to happen. During the surgery, a nurse is close to Daniel all the time, so he is able to ask questions and feel included and seen in the conversations among the staff. Before discharge, Daniel and his husband received information about how the surgery went and about the postoperative period.

### Borderline case

James is having a cholecystectomy. James has suffered with gallstones for years, but his decision to have surgery was only made a few days ago at a visit with his family doctor. During that appointment, James had time to ask questions and the doctor and he had time to talk about the decision to have surgery, both its advantages and disadvantages. On the day of the surgery, the staff appears to be competent and supportive. James trusts the staffs’ competence and feels in control. James is being spoken to directly by the staff prior to the induction of anaesthesia, and up until that point, he is maintaining eye contact with a nurse. However, at the post anaesthesia care unit James feel abandoned. He has nurses around him, but when he wants his wife present to keep him company and to help him remember information provided, that is not allowed due to regulations at the unit.

### Related case

Hanna was recently diagnosed with breast cancer, and she is scheduled for a mastectomy. It has been just a few days since Hanna got her diagnosis, and since then, everything has been happening quickly. She received information about her diagnosis at an appointment, from which she does not remember anything. She does not feel like she had the opportunity to ask any questions, and she is not sure why she is undergoing this procedure today. At the surgery unit, the staff do not seem to have any answers, and they are not able to tell Hanna what the next step after surgery is. Furthermore, the staff seem unsure about the routines for this procedure, and the nurse repeatedly excuse her uncertainty. Hanna is nervous and has a lot of questions, and the staff are perceived as being close, they show empathy for Hanna’s situation, and they do not feel stressed.

### Contrary case

Susan is having shoulder surgery. She is afraid of being anesthetized and wants to be awake during the procedure. Upon arrival at the surgery unit, the staff convince her to undergo general anaesthesia, even though Susan has heard that this type of surgery is performed with regional anaesthesia in other surgery units. She is not provided with any explanations on why regional anaesthesia is not possible for her case. Susan is left alone in the waiting hall for a long time, and nobody can provide her with sufficient information about the schedule and about what is about to happen. The staff she meets at the surgery are perceived as being stressed, and Susan does not know whom to turn to when she has questions. Before the induction of the anaesthesia, Susan feels like the staff are talking over her head, and she falls asleep feeling out of control. When the surgery is completed and Susan is about to be discharged, she wants to have her husband present when she talks with the surgeon, but due to regulations, relatives are not allowed to be present. As Susan has had general anaesthesia, she is tired, and she does not remember what the surgeon said, nor does she remember what the staff said about postoperative medications and about the recovery time.

### Antecedents

According to Walker and Avant (2011), antecedents are the events or incidents that need to take place before the concept occurs. The primary antecedents that need to be fulfilled before patients feel safe in a perioperative context are Knowledge and Relationship. In order to be able to participate, patients should be provided with easy-to-understand information and receive explanations on why certain actions should be carried out (Aasa et al., 2013; Haapala et al., 2013; Hestdal & Skorpen, 2020; Lingehall et al., 2015; Mako et al., 2016; Rosén et al., 2008). Knowledge might be facilitated by information on how the department looks and by the opportunity to familiarize oneself with the environment and to meet the staff beforehand (Aasa et al., 2013; Bergman et al., 2012; Haapala et al., 2013). The staff may help the patient feel supported, cared for, and in control around getting help when needed by providing proper information beforehand about what is about to happen (Bergman et al., 2012; Tosuner Akpinar et al., 2019), about when things like timetables change (Haapala et al., 2013), or about how to handle pain (Adelani & Barrack, 2019). Knowledge from previous experience could also increase the sense of control (Rosén et al., 2008).

A relationship with the staff promotes patients’ feelings of safety and helps them realize that they do not need to be in complete control of the situation and that they are not alone (Barthelsson et al., 2003; Gustafsson et al., 2010; Lindwall et al., 2003; Renholm et al., 2014). Patients have described a wish to have personal preoperative conversations with, e.g., the surgeon, in order to obtain accurate information and be able to ask questions (Dixon et al., 2015; Haapala et al., 2013; Hestdal & Skorpen, 2020. Such a relationship promotes communication (Dixon et al., 2015), as the attitude and empathy of the staff are important for patients (Hommel et al., 2012). One way of promoting communication between the patient and the staff is continuity of care, which might improve knowledge and facilitate participation (Barthelsson et al., 2003; Gustafsson et al., 2010; Renholm et al., 2014). In contrast, if a patient feels like the nurses are not listening to their specific needs, the feeling of security decreases (Costa, 2001), and patients may feel neglected (Larsson et al., 2022).

### Consequences

Consequences are the outcomes of the concept (Walker & Avant, 2011). The literature establishes the consequences of the concept of feeling safe in a perioperative context as Feeling Acknowledged and Trust. Feeling acknowledged involves patients’ desire to be seen as a person, not just a patient (Kaptain et al., 2019). By having a nurse anaesthetist close by and by being able to participate in their own care, patients feel safe and thus feel seen and acknowledged (Bergman et al., 2012; Hestdal & Skorpen, 2020; Larsson et al., 2022). Nurse anesthetists play an important role in making patients feel acknowledged, as they are the person closest to the patient; however, if the patient feel like the nurse anaesthetist is distant, this causes feelings of not being acknowledged in patients (Karlsson et al., 2012), and patients might feel forgotten (Kaptain et al., 2019).

If the staff are perceived as competent and experienced and if patients are able to participate, the patient feels safe (Davis et al., 2013; Heine et al., 2004; Lindwall et al., 2003) and can trust the staff when they take over the responsibility of the patient’s body (Engström et al., 2017). If patients feel like they are in safe hands, their feelings of trust increase (Gustafsson et al., 2010). Furthermore, if patients feel that the staff are competent and experienced and in control, and if they are provided with enough information, patients’ confidence levels increase, which helps them deal with any postoperative complications that might occur (Heine et al., 2011). By participating and by feeling supported, and by feeling in control, patients may have help in coping, which may facilitate their recovery and their regaining of independence (Karlsson et al., 2012; Lingehall et al., 2015; Mako et al., 2016; Turesson et al., 2019).

### Empirical referents

The final step in the concept analysis process of Walker and Avant (2011) is to determine the empirical referents. Empirical referents are a way of recognizing or measuring the defining attributes of a concept, thereby determining its existence. Thus, the empirical referents for feeling safe should comprise indicators that measure the previously-established defining attributes of feeling safe. As described by Mollon (2014), the validation of a patient’s perception of feeling safe may be difficult, due to the fact that feelings are subjective and that perceptions may change. One instrument measuring the perceived feeling of safety in patients undergoing surgery with regional anaesthesia is available (Larsson et al., 2021). The items in its questionnaire were identified from the results from a systematic review (Wassenaar et al., 2014) that found the themes nursing care, patients’ issues, relatives, and technological support were most important for patients’ perception of feeling safe in an intensive care unit, with the most important factor being nursing care. Even though these themes differ from the attributes in this analysis, similarities can be seen; for instance, the importance of a personal approach, information, being in control and included, as well as the presence of nurses, corresponds to the attributes of this analysis. Thus, this instrument may be used as one way to evaluate the perception of feeling safe perioperatively.

Person-centred perioperative care leads to feelings of being respected as a unique person, with one’s needs taken into consideration; thus patients feel involved (Arakelian et al., 2016). One way of measuring patients’ perceptions of person-centred care has been proposed by Fridberg et al. (2020), who developed and evaluated an instrument measuring patients’ perceptions of person-centred care. Evaluating person-centred care may be another way to evaluate patients’ perceptions of feeling safe.

## Discussion

**Figure 2.**
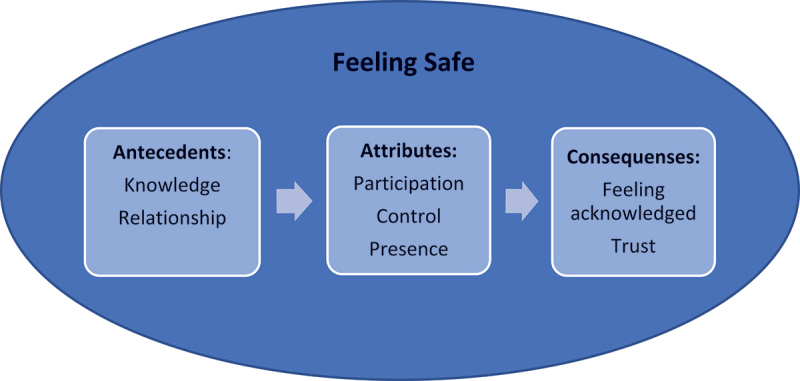
Antecedents, attributes, and consequences of feeling safe in a perioperative setting.

This concept analysis examines the concept of feeling safe from the patient perspective in a perioperative setting and contributes to the development of the concept of feeling safe (Figure 2). The concept of feeling safe is an important part of patient safety work that, as stated by the World Health Organization (n.d.), should be a priority in health care.

This is one of the first studies that explores the concept of feeling safe in this particular (i.e., perioperative) context. Nevertheless, previous studies have been conducted to explore the concept of feeling safe in healthcare contexts. For instance, Mollon (2014) defined the concept for hospitalized patients. Furthermore, other studies have been conducted with the aim of exploring which factors may contribute to patients’ perceptions of feeling safe as well as patients’ experiences of feeling safe. Lasiter (2011) and Wassenaar et al. (2014) studied feeling safe in an intensive care context. Lasiter and Duffy (2013) studied perceived feelings of safety for older patients in both rural and urban acute care. Kenward et al. (2017) explored the factors that contribute to patients’ feelings of unsafety in healthcare settings.

In this study, the attributes of feeling safe are established as Participation, Control, and Presence. Péculo-Carrasco et al. (2020) conclude that person-centred care, together with information and communication, have the most impact on the perceived feeling of safety during prehospital emergency care. In line with this study’s result, the important aspects of person-centred care and feeling safe include the presence of healthcare staff and being treated “humanely” (Péculo-Carrasco et al., 2020, p. 4725). For patients treated at a psychiatric inpatient clinic, the perceived feeling of safety is influenced by a predictable, supportive environment with staff who are communicative and who take time to talk to the patients (Pelto-Piri et al., 2019). Despite its different context, this study is in line with the attributes identified in our study. As suggested above, one way of measuring patients’ perceptions of feeling safe is evaluating person-centred care in certain contexts. With person-centred care, the importance of knowing the person behind the patient is highlighted (Ekman et al., 2011). The relationship between the caregiver and the patient considers their narrative and their thoughts, values, and preferences, which is a crucial component of person-centred practice (Ekman et al., 2011). Thus, person-centred care could make patients feel a greater sense of participation, control and presence, that is, feeling safe in a perioperative context. By defining antecedents, attributes, and consequences of feeling safe, the development of pre-existing theories and models in traditional patient safety work through the incorporation of patients’ perspectives is made possible. This could broaden the traditional view on patient safety and thus promote patient safety work.

Person-centred care practice may be linked to the “little ethics”, termed by the French philosopher Paul Ricoeur (Ekman, 2022). In short, Ricoeur’s “little ethics” (Ricoeur, 2011) is defined as: “aiming for the good life, with and for others in just institutions” (Ricoeur, 2011, p. 68). Ekman (2022) created an adapted definition to fit it to a healthcare context; this adaptation states: “Aiming for health and wellbeing with and for patients, relatives and staff in just institutions” (Ekman, 2022, p. 2). In a perioperative context, there sometimes is an opinion that due to the efficient, highly technological environment, person-centred care is hard to incorporate (Nilsson, 2019). Nevertheless, patients’ perceptions of feeling safe may increase with person-centred care (Ekman et al., 2020). Nurses in the perioperative setting have the important task of being present, both emotionally and physically, to promote the perception of person-centred care (Arakelian et al., 2016). This concept analysis concludes that patients undergoing surgery feel safe when they feel that they participate in their care, that they are in control, and that both healthcare staff and relatives are present. This may be linked to the definition of person-centred care: “health and well-being *with and for* patients, relatives and staff” (Ekman, 2022, p. 2, emphasis added). Thus, person-centred care could increase patients’ sense of safety.

## Strengths and limitations

A limitation of this study is that it lacks a systematic assessment of the quality of the included publications. However, because the goal of this paper’s examination of the literature was to investigate how the concept was used—and not to present evidence of feeling safe in a perioperative context—a critical assessment of the quality of the included studies was deemed unnecessary.

The inclusion criteria in this analysis may have resulted in bias, as the analysis included only peer-reviewed literature that was written in English; therefore, relevant articles might have been missed. Furthermore, there is a risk that alternate terms for “feeling safe” other than those included in this search might have led to the exclusion of potentially relevant articles. However, in order to minimize the risk of this bias, potential keywords for “feeling safe” were identified before the systematic literature search.

## Conclusion

Promoting patients’ sense of safety in a perioperative setting is an important part of nursing. This concept analysis has identified the defining attributes of the concept in this specific context. By defining the concept of feeling safe in a perioperative setting, this study provides an opportunity to develop already-existing theories and models. This concept analysis underscores the importance of including patients’ perceptions in traditional patient safety work. Person-centred care could be one possible way of evaluating patients’ perceptions of feeling safe, as well as increasing the perceptions of feeling safe of people who undergo surgery. Patients who feel safe perceive that they participate in their care, that they are in control, and that they feel the presence of both healthcare staff and relatives. The perceived feeling of security could, by extension, promote the postoperative recovery of patients undergoing surgery.

## Data Availability

The datasets generated and analysed during the current study are available from the corresponding author upon reasonable request.
